# How does the import of environmental intermediate goods affect CO_2_ emissions? theoretical and empirical research of prefecture-level cities of China

**DOI:** 10.1371/journal.pone.0290333

**Published:** 2023-08-31

**Authors:** Wenhua Yuan, Weixiao Lu, Junyan Zhang

**Affiliations:** 1 Institute of International Economy, University of International Business and Economics, Beijing, China; 2 Business School, Yangzhou University, Yangzhou, Jiangsu, China; 3 Institute of Economics, Shanxi University of Finance and Economics, Taiyuan, China; Guangzhou Institute of Geography, Guangdong Academy of Sciences, CHINA

## Abstract

This paper constructs a theoretical model of biased production decisions due to the import of environmental intermediate goods. Additionally, it analyzes the influence of these imports on CO_2_ emissions based on the trade and CO_2_ emission data of Chinese prefecture-level cities from 2000 to 2016. Furthermore, it empirically explores how environmental intermediate imports affect CO_2_ emissions. The study found the following: first, the import of environmental intermediate goods can effectively reduce CO_2_ emissions; this conclusion still holds under robustness and endogeneity tests. Second, the carbon emission reduction effect related to the import of environmental intermediate goods is affected by differences in geographical location, environmental pollution, sustainable development ability and government efficiency. Third, the mechanism test found that the import of environmental intermediate goods exerts emission reduction effects through the green technological innovation and industrial structure upgrading mechanisms. The conclusions of the research in this article provide a reference for coordinating trade development and environmental protection.

## 1. Introduction

In the increasingly challenging context of global environmental degradation and economic globalization, trade-implied CO_2_ and international CO_2_ leakage are receiving increasing attention in academia and in international climate negotiations. International trade is an important means of promoting economic growth and of participation in global value chains. However, it also entails international trade-induced embodied CO_2_ flows, i.e., the transfer of CO_2_ emissions across spatially separated regions. Balancing the economic and environmental benefits associated with open trade is an area of focus in social governance and academic research in various countries.

Developed countries shift pollution-intensive industries to developing countries and then import pollution-intensive products from them [1]. This process increases pollution emissions in developing countries and is known as the pollution paradise hypothesis (PHH) [2]. Research based on the PHH that focuses on the trade in manufactured goods indicates that product importation naturally reduces domestic CO_2_ emissions as the related production processes are shifted abroad. While this view has some merit, it ignores the feature of the import trade in which intermediate goods account for a large share, i.e., the processing re-export trade. In recent decades, there has been a strong tendency toward the fragmentation of production, and the intermediate goods trade has seen significant growth [3]. The intermediate goods trade is an important part of international trade, and an increase in the trade in import intermediate goods leads to an increase in the scale of local upstream and downstream industries, which in turn leads to an increase in the export trade in final goods. The expansion of intermediate trade also means the expansion of final trade, which may expand the scale of local polluting industries and worsen the regional ecological environment. At the same time, the PHH fails to effectively analyze the dynamic changes in the level of production technology and the industrial structure of each country. With the development of various environment-related emerging industries, current research needs to assess the influence of the fast-growing trade in intermediate goods more broadly.

Environmental intermediate goods are intermediate products used to measure, prevent, control, mitigate, and remediate various environmental problems [4]. In the 2001 Doha Round negotiations, the World Trade Organization (WTO) singled out environmental intermediate goods and advocated for reducing the trade barriers for such goods to promote the global flow of low-CO_2_ environmental products and expand the dissemination of environmental technologies. These actions were designed to enhance the ability of countries to address climate change and resolve environmental issues. Since then, the environmental intermediate goods trade has become a major factor in coordinating open trade and low-CO_2_ development and in providing opportunities to address global climate change and deepen global value chains.

However, there are more questions about whether the trade in environmental goods can truly promote low-CO_2_ development, and the effectiveness of such trade has not yet been realistically tested. The promotion and use of renewable energy are thought to directly contribute to reducing greenhouse gas emissions, and the environmental intermediate goods trade theoretically includes many renewable energy-related products. This trade can drive the development of green industries in trading countries (especially in exporting countries), thus improving resource productivity and boosting green economic development.

However, in practice, there is still much uncertainty about the influence of the environmental intermediate goods trade on regional CO_2_ emissions. First, the relatively high technical thresholds of the environmental intermediate goods trade, the high initial investment, the slow diminishing returns, and the high investment risks and uncertainties have severely limited the willingness of developing countries to engage in this type of trade [5]. Second, in the environmental trade process, the status of trading partner countries is not equal; different countries benefit differently from the trade process. This situation is likely to result in trade gains accruing to developed countries [6], while developing countries are not necessarily able to reap environmental benefits. On the one hand, this imbalance may weaken the incentive for developing countries to participate in the environmental intermediate goods trade; on the other hand, it may encourage developed countries to further erect green trade barriers and hinder the global diffusion of low-carbon green technologies. Finally, the degree of a country’s competitiveness in the environmental intermediate goods trade, its position in the global trade network, and the differences in regional endowments may affect the CO_2_ emission reduction effect. Therefore, great theoretical significance and practical value can be gained from studying the influence of environmental intermediate goods imports on CO_2_ emissions, clarifying the associated theoretical mechanism, and using the environmental intermediate goods trade to promote low-carbon green development to countries worldwide.

China’s distinctive development history provides an excellent case study of how the import of environmental intermediate goods affects CO_2_ emissions. Since China joined the WTO, its position in the global trade network has improved significantly, and its scale of imports has expanded, with the intermediate goods trade taking a growing share. According to UN COMTRADE statistics, since China joined the WTO in 2021, imports of semifinished goods have accounted for 70% of total merchandise imports. Semifinished goods have become the largest import category. Meanwhile, China’s CO_2_ emissions have been increasing; currently, China is ranked first worldwide in both total trade and CO_2_ emissions. The Chinese government’s 14th Five-Year Plan proposes the creation of a green trade development platform, the achievement of substantial goals in regard to CO_2_ peaking and CO_2_ neutrality, the steady promotion of green trade development, reductions in CO_2_ emission intensity, and adherence to the implementation of a broader and deeper opening up. Furthermore, the plan proposes to strengthen the dual cycle of mutual promotion and integration of domestic and international cycles, actively expand imports, implement high-level openness, and create a new state of win-win cooperation. Actively expanding imports, building a new dual cycle development pattern, strengthening environmental protection and achieving the dual carbon goals have become important poles in China’s current economic work.

On this basis, this paper constructs a theoretical model for the biased production decisions of enterprises. Based on the theory of biased technological progress, we theoretically analyze how the imports of environmental intermediate goods affect the relative demand for production factors, technological progress, and the dynamic change in the production structure of enterprises. Subsequently, using the Chinese customs database and the CO_2_ emission database of Chinese prefecture-level municipalities and taking the products included in the Organization for Economic Cooperation and Development (OECD) Consolidated List of Green Intermediate Goods (CLGE) as environmental intermediate goods, we construct an index of the import openness of prefecture-level municipalities in China to environmental intermediate goods. Next, we empirically examine how environmental intermediate goods imports affect CO_2_ emissions in importing regions and further analyze the specific mechanism of this emission reduction effect and the heterogeneity of different scenarios. Additionally, this paper explores the spatial spillover effect of imported environmental intermediate goods on CO_2_ emissions. This study provides theoretical support and a practical basis for deepening the understanding of trade openness and low-carbon economic development. Furthermore, it helps realize green low-carbon development goals through the structural optimization and quality improvement of imported intermediate goods. The clarification of the effect mechanism provides a reference for future governments to formulate development plans.

## 2. Literature review

Regarding the influence of the import trade on CO_2_ emissions, some scholars believe that the import trade increases CO_2_ emissions. The signing of the Kyoto Protocol promoted pollution transfer. As developing countries continued to develop polluting industries and process imported raw materials, their CO_2_ emissions increased significantly [4]. Based on trade data on the import of intermediate goods from emerging economies, some scholars have found that per capita CO_2_ emissions and emission intensity increase as the scale of imports increases [7]. Some scholars have proposed a causal relationship between imports and CO_2_ emissions in terms of foreign exchange policy and international investment [8,9].

Sectoral scholars believe that the import trade reduces CO_2_ emissions. Some scholars argue that vertical trade can reduce pollution through the spillover of green technologies and that there is a negative correlation between imports and CO_2_ emissions based on trade and environmental data from several developing countries [10,11]. The substitution and technological upgrading effects of the import trade reduced CO_2_ emissions from manufacturing in Australia, which is essential for environmental improvement [12]. The import trade improves the productivity of firms in importing countries through technological spillovers, and thus, it reduces CO_2_ emissions [13]. Under the China-Japan-Korea free trade agreement (FTA), it was found that increased import openness can induce countries to exploit their resource endowments and adjust their industrial structures to jointly achieve CO_2_ emission reductions [14].

Sectoral scholars argue that the influence of imports on CO_2_ emissions is nonlinear and does not have a uniform pattern. Some scholars have relied on the environmental Kuznets curve (EKC) hypothesis, which states that there is an inverted U-shaped relationship between emissions of environmental pollutants such as CO_2_ and income in that emissions rise and then fall as per capita income rises and imports are positively correlated with per capita income levels. Therefore, they believe that imports and CO_2_ emissions may also have an inverted U-shaped relationship. The CO_2_ emissions of developed countries have a stable negative relationship with foreign trade, while the relationship of these emissions with foreign trade in developing countries is not clear [15]. There may be a threshold in the environmental influence of trade, with trade increasing CO_2_ emissions when it is below the threshold and decreasing CO_2_ emissions when it is above the threshold [16]. Another group of scholars believes that the import trade and CO_2_ emissions have no uniform pattern between them due to individual or spatiotemporal heterogeneity, especially in China. The import trade has a positive influence on environmental improvement in Eastern China for regions that increased import openness, while it has a negative impact in Central and Western China [17]. The growth of total imports increases environmental pollution in the short term, but the influence of imports on CO_2_ emissions is not significant in the long term [18].

Relatively few studies have considered the influence of the intermediate goods trade on regional CO_2_ emissions. These studies have focused on empirical work in the field of energy efficiency. Few studies have focused on the influence of the intermediate goods trade on CO_2_ emissions. There is a moderating effect of the intermediate goods trade on the relationship between energy consumption and CO_2_ emissions, and it was found that the intermediate goods trade helps reduce CO_2_ pollution [19]. Using firm-level Indonesian data, researchers found that firms that import intermediate goods consume 13.9% less energy than nonimporting firms [20]. The intermediate goods trade is an important source of CO_2_ emission mitigation, and there is a unidirectional causal relationship between the intermediate goods trade and CO_2_ emissions [21]. The diversification of intermediate goods imports has a significant negative influence on CO_2_ emissions in developed countries but a positive influence in developing countries [22]. Intermediate goods imports can promote urban energy efficiency, for which technological spillovers and the diversification of intermediate product types are the key mechanisms [23].

In summary, the literature includes in-depth studies on the relationship between imports and CO_2_ emissions and preliminary explorations of how the importation of intermediate goods affects regional CO_2_ emissions. However, these explorations still need to be expanded in the following two way: (i) in terms of theory, traditional studies mostly focus on the substitution effect of the final goods import trade, while some of the literature focuses on technological spillovers. However, most empirical studies lack theoretical models. Targeted mathematical and theoretical models have not been established to determine how the import of environmental intermediate goods impacts the biased production decisions and the research and development (R&D) technology of firms, which in turn lead to endogenous CO_2_ emission reduction effects. (ii) In terms of empirical evidence, the literature focuses on industry- or country-level data; relatively few studies use micro data at the city level and examine spatial spillovers. Moreover, the literature mostly uses the total imports and exports of all intermediate goods to measure the regional import trade in intermediate goods without effectively distinguishing the characteristics of specific environmental intermediate goods. Further refinement of empirical research data is necessary to construct more targeted indicators.

The possible marginal contributions of this article are as follows: (i) the research perspective explores the biased production decisions caused by the import of environmental intermediate goods and the resulting environmental protection effects, and the study constructs a rigorous and objective theoretical model to deepen and extend the existing research perspective in the literature. (ii) Based on China’s customs database, nighttime light data, energy consumption data, and terrestrial vegetation CO_2_ sequestration data, this study accurately measures the import value of environmental intermediate goods and CO_2_ emissions for prefecture-level municipalities, as the aforementioned data are more accurate and reliable indicators of this import value. (iii) This paper studies not only the emission reduction effect and mechanism of local and current environmental intermediate goods imports but also the related spatial spillover effect, thus providing more detailed and comprehensive research.

## 3. Theoretical analysis

To identify the theoretical basis of the CO_2_ reduction effect of environmental intermediate goods imports, we construct a theoretical model for the biased production decisions of firms related to environmental intermediate goods imports [5].

### 3.1 Demand

Assuming the product market is monopolistically competitive, the demand function faced by firm *i* is the standard Dixit–Stiglitz form of the monopolistic competition model:

$$y_{it}=\phi_{it}\Phi_{t}p_{it}^{-\eta }$$ (1)

where *ϕ*_*it*_ is the firm-specific demand coefficient, which represents the heterogeneity of firm demand; *Φ*_*t*_ is the industry demand coefficient; *p*_*it*_ is the firm’s product price; and *η* > 1 is the constant elasticity. In addition, *i* and *t* indicate the firm and year, respectively.

In a monopolistic competition market structure, firm *i* sets the sales price at a constant markup rate on the marginal cost, and thus, the firm’s product price is as follows:

$$p_{it}=\left(\frac{\eta }{\eta -1}\right)\;c_{it}$$ (2)

where *c*_*it*_ is the marginal cost of production of the firm.

### 3.2 Production

We introduce environmental intermediate goods into the manufacturing production process and assume that the production process requires the input of three production factors: labor (*l*), capital (*k*), and environmental intermediate goods (*G*). The production function is in Cobb-Douglas form as follows:

$$y_{it}=k_{it}^{\alpha_{k}}l_{it}^{\alpha_{l}}G_{it}^{\gamma }e^{{\tilde{v}}_{it}}$$ (3)

where *α*_l_, *α*_*k*_ and *γ* are the output elasticities of labor, capital, and environmental intermediate goods, respectively; ${\tilde{v}}_{it}$ is the Hicks-neutral factor of the production coefficient. Capital inputs are fixed in the short run, and labor and environmental intermediate goods inputs are variable in the short term.

According to the cost minimization principle, the short-term marginal cost function of firm *i* is as follows:

$$c_{it}=\varphi_{1}y_{it}^{\frac{1-\alpha_{l}-\gamma }{\alpha_{l}+\gamma }}k_{it}^{-\frac{\alpha_{k}}{a_{l}+\gamma }}w_{t}{}^{\frac{\alpha_{l}}{\alpha_{l}+\gamma }}P_{it}^{g}{}^{\frac{\gamma }{\alpha_{l}+\gamma }}{\text{e}}^{-\frac{1}{\alpha_{l}+\gamma }\tilde{v_{it}}}$$ (4)

where $\varphi_{1}\equiv \alpha_{l}^{-\frac{\alpha_{l}}{\alpha_{l}+\gamma }}\gamma^{-\frac{\gamma }{\alpha_{l}+\gamma }}$ is a constant. *w*_*t*_ is the factor price of labor. $P_{it}^{g}$ is the factor price of environmental intermediate goods.

From the demand function (1) and product price function (2) faced by the firm, the revenue function of firm *i* can be obtained as follows:

$$r_{it}=\varphi_{2}\Phi_{t}^{\frac{1}{\tau }}k_{it}^{-\left(\alpha_{k}\right)\frac{\left(1-\eta \right)}{\tau }}w_{t}{}^{\left(-\alpha_{k}\right)\frac{\eta -1}{\tau }}P_{it}^{g}{}^{\left(-\gamma \right)\frac{\eta -1}{\tau }}{\text{e}}^{v_{it}}$$ (5)

where $\varphi_{2}={\left(\alpha_{l}^{-\alpha_{l}}\alpha_{s}^{-\gamma }\right)}^{\frac{1-\eta }{\tau }}{\left(\frac{\eta }{\eta -1}\right)}^{\frac{1-\eta }{\tau }\left(\alpha_{l}+\gamma \right)}$, $\tau \equiv 1+\left(1-\alpha_{l}-\gamma \right)\left(1+\eta \right)$, *τ* > 1, and $v_{it}=\frac{1}{\tau }\left[\text{ln}\phi_{it}+\left(\eta -1\right){\tilde{v}}_{it}\right]$. *v*_*it*_ is the indication of firm heterogeneity. The larger the firm-specific demand coefficient *ϕ*_*it*_ and the higher the firm-specific production efficiency ${\tilde{v}}_{it}$ are, the stronger the manifestation of firm heterogeneity characteristics.

According to the Dixit–Stiglitz model of monopolistic competition, firm profits are proportional to revenues:

$$\pi_{it}=\left[1-\frac{\eta -1}{\eta }\left(\alpha_{l}+\gamma \right)\right]r_{it}=\varphi_{3}\Phi_{t}^{\frac{1}{\tau }}k_{it}^{-\left(\alpha_{k}\right)\frac{\left(1-\eta \right)}{\tau }}w_{t}{}^{\left(-\alpha_{k}\right)\frac{\eta -1}{\tau }}P_{it}^{g}{}^{\left(-\gamma \right)\frac{\eta -1}{\tau }}{\text{e}}^{v_{it}}$$ (6)

where $\varphi_{3}=\varphi_{2}\left[1-\frac{\eta -1}{\eta }\left(\alpha_{l}+\gamma \right)\right]$ is a constant.

The total amount of *G*_*it*_ used by *i* is the set of all types of intermediate goods *g*_*it*_. Following the method of existing research [13], the total amount of environmental intermediate goods input *G*_*it*_ can be obtained from the production function as follows:

$$G_{it}={\left(\int_{j\in J}g_{j}^{*}{}^{\left(1-\sigma \right)/\sigma }dj\right)}^{\frac{\sigma }{1-\sigma }}$$ (7)

where *j* = 1,2,……., *J* is the set of environmental intermediate goods.

It is assumed that the price of environmental intermediate goods is influenced by import trade openness, and thus, the higher the import trade openness is, the larger the quantity of imported environmental intermediate goods and the larger the set of options available to domestic firms. As a result, the more competitive the supply market of environmental intermediate goods is, the lower the factor price. By combining the price index with the production function, the total price of environmental intermediate goods $P_{it}^{g}$ can be obtained as follows:

$$P_{it}^{g}={\left(\int_{j\in J}\;\mu p_{ijt}^{g}{{}^{*}}^{1-\sigma }dj\right)}^{\frac{1}{1-\sigma }}$$ (8)

where *μ* = *τ*^1−*σ*^ represents the import openness to environmental intermediate goods and 0 < *μ* ⩽ 1. *τ* > 1 refers to the iceberg transport cost, and $p_{ijt}^{g}{}^{*}$ denotes the import price of various environmental intermediate goods, which is exogenously determined by overseas markets.

Furthermore, from Eq (8), we can obtain the following:

$$\frac{\partial P_{it}^{g}}{\partial \mu }<0$$ (9)

That is, greater import openness reduces the price of environmental intermediate goods in the domestic market, enhances green R&D capabilities and technological innovation, and ultimately reduces the comprehensive production costs of firms.

### 3.3 Production decision

#### 3.3.1 Two modes of intermediate goods processing activities

After acquiring intermediate goods, firms produce different types of final goods by reprocessing them and combining them with other elements. In the process of processing intermediate goods to produce final goods, firms can choose either high-carbon processes with low technical requirements and low value added, thus producing high-carbon final goods, or low-carbon processes with high technical requirements and high value added, thus producing low-carbon final goods. The heterogeneity of a firm’s final products (*v*_*it*_) mainly depends on its choice of processing methods. Drawing on the approach of existing research, the heterogeneous characteristic performance of firms is assumed to follow a first-order Markov process:

$$v_{it}^{T}=\lambda_{0}+\lambda_{1}v_{it-1}+\lambda_{2}{\left(v_{it-1}\right)}^{2}+u^{T}\left(q_{it-1}^{T}\right)+\xi_{it}\left(T\in \left\{L,S\right\}\right)$$ (10)

where *L* and *S* denote high-carbon and low-carbon processing, respectively. $q_{it-1}^{T}\left(T\in \left\{L,S\right\}\right)$ denotes the processing mode selection of firm *i* in period *t* − 1 and satisfies 0 < *u*^*L*^(⋅) < *u*^*S*^(⋅), 0 < *u*^*L*′^(⋅) < *u*^*S*^′(⋅), 0 < *u*^*L*″^(⋅) < *u*^*S*″^(⋅), *u*^*L*^(0) = 0 and *u*^*S*^(0) = 0. *ξ*_*it*_ denotes a random shock.

The cost functions of a firm’s high-carbon and low-carbon processing methods are as follows:

$$n_{it}^{L}\left(q_{it}^{L}\right)=h^{L}\left(q_{it}^{L}\right)d^{L}\left(G_{it}\right)$$ (11)

$$n_{it}^{S}\left(q_{it}^{S}\right)=h^{S}\left(q_{it}^{S}\right)d^{S}\left(G_{it}\right)$$ (12)

where *n*^*L*^ and *n*^*S*^ denote the costs of high-carbon and low-carbon processing, respectively. $h^{T}\left(q_{it}^{T}\right)\left(T\in \left\{L,S\right\}\right)$ denotes the fixed costs of the two production methods and satisfy *h*^*T*^(0) = 0(*T* ∈ {*L*, *S*}), 0 < *h*^*L*^(⋅) < *h*^*S*^(⋅) 0 < *h*^*L*′^(⋅) < *h*^*S*′^(⋅), and 0 < *h*^*L*″^(⋅) < *h*^*S*″^(⋅). *d*^*T*^(*G*_*it*_)(*T* ∈ {*L*, *S*}) denotes the technological spillover effect brought by processing with environmental intermediate goods. Since advanced elements such as the technological knowledge contained in environmental intermediate goods are closer to the low-carbon processing process and, therefore, low-carbon processing is more advantageous for absorbing and transforming technological spillovers, this paper assumes that the processing cost reduction effect caused by technological spillovers from environmental intermediate goods imports occurs only in the low-carbon processing process [6], i.e., *d*^*L*^(*G*_*it*_) ≡ 1, *d*^*S*^(*G*_*it*_) < 1 and satisfies *d*^*S*′^(⋅) < 0 and *d*^*S*″^(⋅) < 0.

We assume that the CO_2_ emissions generated during the processing of a firm are as follows:

$$e_{it}^{T}=\varphi \left(\left.q_{it}^{T}\right)k_{it}^{\alpha_{k}}l_{it}^{\alpha_{l}}G_{it}^{\gamma }e^{{\tilde{v}}_{it}}\right.\left(T\in \{L,S\}\right)$$ (13)

$$\varphi \left(q_{it}^{T}\right)={\left(1+\mu \right)}^{-\beta /1-\beta }{\left(1-q_{it}^{T}\right)}^{1/1-\beta }\left(T\in \{L,S\}\right)$$ (14)

where $\varphi \left(q_{it}^{T}\right)$ indicates the CO_2_ emission reduction effect caused by using environmental intermediate goods in the production process and satisfies 0 < *β* < 1. With an increase in openness in terms of importing environmental intermediate goods, the quality of the intermediate elements used by enterprises for production is improved, making the production process cleaner and thus reducing CO_2_ emissions. The higher the proportion of low-carbon processing is, the more obvious the emission reduction effect is, i.e.,

$$e_{it}\left(q_{it}^{S}\right)<e_{it}\left(q_{it}^{L}\right)$$ (15)

#### 3.3.2 Production decision and expected profit

Which processing activity a firm chooses and what type of final product it produces are related to the revenue generated by different processing modes. Therefore, the firm’s production decision problem can be expressed as follows:

$$\begin{array}{c} V\left(v\right)=\pi_{it}\left(v_{it}\right)+{\text{max}}_{q_{it}^{L}⩾0,q_{it}^{s}⩾0}\left\{\theta^{L}E\left[V^{L}\left(v_{it+1}^{L}|v_{it},q_{it}^{L}\right)\right]-h^{L}\left(G_{it}^{L}\right)\times 1,\right.\\ \left.\theta^{S}E\left[V^{S}\left(v_{it+1}^{S}|v_{it},q_{it}^{S}\right)\right]-h^{S}\left(q_{it}^{S}\right)d\left(G_{it}\right)\right\} \end{array}$$ (16)

where *θ*^*T*^(*T* ∈ {*L*, *S*}) indicates the probability that the firm chooses this type of processing and satisfies 0 < *δ*^*S*^ < *δ*^*L*^, which is mainly due to the lower technical requirements of the high-carbon process and, thus, the higher probability of being selected under the same conditions.

Since Eq (16) cannot be solved directly, we analyze it in terms of marginal benefits and marginal costs. From the Markov process Eq (10) and the profit function Eq (6), the expected profit of firm $E\left[\pi_{it+1}^{T}\left(v_{it},q_{it}^{T}\right)\right]\left(T\in \left\{L,S\right\}\right)$ and the partial derivative of the import openness to environmental intermediate goods *μ* can be derived as follows:

$$\frac{\partial \left\{\frac{\partial E\left[\pi_{it+1}^{T}\left(v_{it},q_{it}^{T}\right)\right]}{\partial q_{it}^{T}}\right\}}{\partial \mu }>0,T\in \left\{L,S\right\}$$ (17)

This calculation suggests that the higher the import openness to environmental intermediate goods is, the lower the production costs and the higher the expected profits, which in turn will motivate firms to further increase their imports of environmental intermediate goods.

In addition, the derivation of the low-CO_2_ processing cost function of the firm with respect to the import openness to environmental intermediate goods *μ* shows the following:

$$\frac{\left\{\frac{\partial \left[h^{S}\left(q_{it}^{S}\right)d\left(G_{it}\right)\right]}{\partial q_{it}^{S}}\right\}}{\partial \mu }=h^{S^{\prime }}\left(q_{it}^{S}\right)\times d^{\prime }\left(G_{it}\right)\times G_{it}^{\prime }\left(p_{it}^{g}\right)\times p_{it}^{g^{\prime }}\left(\phi \right)<0$$ (18)

In view of Eq (15), the more open the market is to the import of environmental intermediate goods, the lower the factor price of environmental intermediate goods in the domestic market, the more substantial the complementarity with low-carbon production processes, the lower the production costs of low-carbon processing processes, and the higher the expected profits of enterprises, which will motivate enterprises to choose more low-carbon processing processes and ultimately achieve CO_2_ emission reductions.

Based on the theoretical analysis above, this article assumes the following:

**Hypothesis 1**: Increased openness to imports of environmental intermediate goods can effectively contribute to reducing CO_2_ emissions.
**Hypothesis 2**: Increased openness to imports of environmental intermediate goods effectively reduces CO_2_ emissions by increasing the green technological innovation ability of firms.
**Hypothesis 3**: Increased openness to imports of environmental intermediate goods effectively reduces CO_2_ emissions by promoting industrial structure optimization and upgrading.

## 4. Methods and data

### 4.1 Method of estimation

To verify the relationship between the import of environmental intermediate goods and CO_2_ emissions, we set up the following formal econometric model:

$$Y_{it}=\alpha_{0}+\alpha_{1}X_{it}+\alpha_{k}\sum_{k}Control_{k}+\gamma_{i}+\lambda_{t}+\varepsilon_{it}$$ (19)

where the subscript *i* denotes the city, *t* denotes the year, *Y*_*it*_ denotes the CO_2_ emissions of city *i* in year *t*, *X*_*it*_ denotes the import openness to high-value environmental inputs imported by city *i* in year *t*, *Control*_*k*_ denotes *k* control variables, *γ*_*it*_ and *λ*_*t*_ denote city and year fixed effects, respectively, and *ε*_*it*_ is the error term.

### 4.2 Variables

#### 4.2.1 Dependent variable

CO_2_ emissions: In this paper, CO_2_ emission data at the level of prefecture-level cities in China are calculated by summing the emissions of the subordinate counties [24]. First, the particle swarm optimization back propagation (PSO-BP) algorithm is used to unify the Defense Meteorological Satellite Program (DMSP)/Operational Linescan System (OLS) and National Polar-orbiting Partnership (NPP)/Visible Infrared Imaging Radiometer Suite (VIIRS) satellite image scales to calibrate the Chinese nighttime light data. Second, provincial energy CO_2_ emissions are measured using the Intergovernmental Panel on Climate Change (IPCC) methodology and provincial energy balances. Finally, based on the proportional relationship of the nighttime light brightness and the energy consumption of each county to the CO_2_ emission data of the corresponding province, county-specific CO_2_ emissions are again measured to obtain county-level CO_2_ emissions. This method can ensure the accuracy of CO_2_ emissions at the county level to the greatest extent.

Considering that the goal of CO_2_ neutrality is to achieve a dynamic balance between CO_2_ emissions and absorption, we use the CO_2_ sequestration values of terrestrial vegetation at the county level to measure the net CO_2_ emissions of prefecture-level cities as a robustness check [24]. The calculation is as follows: first, taking the MOD17A3 data released by the National Aeronautics and Space Administration (NASA) and multiplying by a 0.0001 conversion factor, we obtain net primary productivity data and then calculate the CO_2_ sequestration value of terrestrial vegetation based on the conversion factor (i.e., 1.62/0.45), including evergreen/deciduous/coniferous forests, mixed forests, open/closed shrublands, grasslands and agricultural crops.

#### 4.2.2 Independent variable

Environmental intermediate goods imports openness (*EIO*): Considering that the research topic of this paper is CO_2_ emission reduction, in this paper, environmental intermediate goods refer to the products included in the OECD CLGE, and the import openness to environmental intermediate goods (EIO) refers to the ratio of the total imports of products included in the OECD CLGE to the GDP of the local municipality in that year. At present, no uniform definition of environmental intermediate goods exists in CO_2_ emission reduction research. However, the WTO has established "The Friends’ List" of green products; the Asia-Pacific Economic Cooperation (APEC) has established a list of environmental intermediate goods containing 54 commodities. the OECD has also established a list of environmental intermediate goods (PEGS) containing 150 commodities. Since then, the OECD has continued to update and improve the CLGE, which covers 248 intermediate goods, and the Core List of Green Intermediates (CLGE+). Hence, this study selects products included in the CLGE, which contains a wide range of commodities that are recognized as environmental intermediate goods [6]. Product-level data were obtained from the EPS data platform and the Chinese customs database.

#### 4.2.3 Control variables

(i) Considering that the EKC hypothesis suggests a dynamic relationship between air quality and per capita income, which presents an inverted U shape that increases first and then decreases, this study introduces per capita GDP (*PGDP*) and the squared term of per capita GDP (*PGDPS*) to control for the effect of changes in per capita income on CO_2_ emissions.
(ii) China is currently in a critical period of urbanization construction. According to international urbanization experience, when the urbanization rate exceeds 70%, economic growth is driven by industrial development and change to rely on technological progress, knowledge spillovers and other innovation factors, and cities enter a sustainable low-carbon development stage. Meanwhile, the development of urbanization means that a large number of rural residents migrate to cities; residents have more opportunities to receive education and training; improvements in human capital ease environmental pollution pressures; increases in human capital lead to cleaner types of production; and sustainable growth ultimately promotes urban CO_2_ emission reductions. Therefore, this paper introduces the urbanization rate (*URB*) and population density (*DEN*) to control for the effects of urbanization and human capital on CO_2_ emissions.
(iii) The industrial structure tends to change gradually from an agricultural-dominated structure to an industrial-dominated structure and then to a service-dominated structure. Moreover, industrial manufacturing has high levels of pollution and high emissions. Industrial structure upgrading is accompanied by service sector increases, which reduce CO_2_ emissions. The adjustment of the industrial structure in different cities leads to dynamic changes in CO_2_ emission intensity. Hence, this study introduces the share of the secondary industry (*STR*) and share of the tertiary industry (*TTR*) to control for the influence of the industrial structure. The industry share is measured using the ratio of industry employment to total city employment.
(iv) According to the PPH, firms in polluting industries tend to relocate to countries with relatively low environmental standards, thereby circumventing environmental regulations and thus undoubtedly increasing environmental pressure. At present, the scale of polluting industries among the industries benefitting from foreign investment in China is relatively large. Thus, this paper introduces the ratio of actual foreign investment to GDP (*FDI*) to control for the influence of foreign investment.
(v) Local governments should strengthen environmental controls and introduce stricter environmental protection measures that force enterprises to use clean technology instead of traditional technology to address the deterioration of the urban environment, increase the intensity of technological innovation, and promote the transformation of enterprises’ production methods to cleaner production. Furthermore, increasing the intensity of environmental governance by local governments and providing financial and fiscal support and policy incentives to enterprises or individuals for new energy and cleaner production can raise the overall awareness of environmental protection efforts and increase investment in environmental governance. Therefore, to control for the influence of environmental protection efforts, we introduce environmental governance intensity (*ENV*), which is measured by the proportion of the amount of investment in pollution treatment to the public budget expenditure of cities.
(vi) The promotion and application of low-carbon technologies can provide cleaner production technology options for enterprise development and effectively promote a reduction in CO_2_ emissions while stabilizing economic growth, with scientific and technological progress being the core and key to developing a low-carbon economy and tackling climate change. Therefore, this study introduces the level of science and technology (*SCI*) to control for the influence of science and technology. *SCI* is measured by the ratio of science and technology employees to the total number of employees in the city.
(vii) Increasing investment in education will increase the reserve of high-level low-carbon talent, improve the skill level of workers, and effectively improve energy utilization efficiency. Moreover, the comprehensive implementation of ecological environmental education, the organic integration of ecological environmental education and subject teaching, and the improvement in the environmental protection education curriculum system with hierarchical connections can effectively enhance the public’s awareness of the need to protect the ecological environment. This awareness is the basic internal driving force for the sustainable development of the low-CO_2_ economy in the future. Therefore, this paper introduces education input intensity (*EDU*) to control for the influence of local education investment on CO_2_ emissions, where urban education input intensity is measured by the proportion of education expenditure to the public budget.

The data for the control variables above were obtained from the China City Statistical Yearbook. The sample period of this paper is from 2000 to 2016, considering that Chinese customs data are available from 2000 to 2016. All variables were treated logarithmically in the empirical process. Table 1 shows the descriptive statistics.

**Table 1:** Descriptive statistics.

Variables	Meaning	Num	Ave	Std	Min	Max
*CO2*	Carbon emissions	4 901	0.184	0.105	0.063	0.374
*EIO*	Environmental intermediate goods import openness	4 901	0.005	0.008	0.000	0.046
*PGDP*	GDP per capita	4 901	3.068	0.641	2.176	4.338
*PGDPS*	Square of GDP per capita	4 901	6.023	1.343	4.128	8.650
*URB*	Urbanization rate	4 901	0.443	0.194	0.094	1.525
*DEN*	Population density	4 901	0.059	0.005	0.052	0.065
*STR*	Share of the secondary industry	4 901	0.372	0.0300	0.315	0.415
*TTR*	Share of the tertiary industry	4 901	0.501	0.140	0.088	0.772
*FDI*	Share of foreign investment	4 901	0.124	0.064	0.003	0.484
*ENV*	Environmental governance intensity	4 901	0.030	0.026	0.000	0.121
*SCI*	Level of science and technology	4 901	0.453	0.051	0.200	0.640
*EDU*	Education input intensity	4 901	0.028	0.032	0.002	0.393

## 5. Results

### 5.1 Baseline

This paper first uses stepwise regression in a baseline model. As shown in Table 2, the estimated coefficients of the regression of the effect of the import openness to environmental intermediate goods on CO_2_ emissions are significantly negative, indicating that imports of environmental intermediate goods significantly reduce CO_2_ emissions. This finding validates hypothesis 1. In recent years, China has been gradually paying attention to the problem of environmental pollution. The type effect, quality effect, and knowledge spillover effect of environmental intermediate goods imports can motivate enterprises to adopt and use clean technologies to reduce production costs and ultimately reduce CO_2_ emissions. These factors increase the enterprise cost of treating waste gas and wastewater, while the type effect, quality effect and knowledge spillover effect of environmental intermediate goods imports can motivate enterprises to introduce and use clean technologies to reduce CO_2_ emissions.

**Table 2:** Baseline regression.

Variables	(1)	(2)	(3)	(4)	(5)	(6)
	CO2	CO2	CO2	CO2	CO2	CO2
*EIO*	-0.559*** (-5.404)	-0.285*** (-2.655)	-0.412*** (-4.417)	-0.398*** (-3.920)	-0.384*** (-3.906)	-0.372*** (-3.837)
*PGDP*		0.327*** (4.071)	0.712*** (9.031)	0.735*** (9.578)	0.618*** (7.352)	0.611*** (7.200)
*PGDPS*		-0.138*** (-3.624)	-0.321*** (-8.560)	-0.334*** (-9.181)	-0.279*** (-7.014)	-0.276*** (-6.865)
*URB*			0.142*** (19.137)	0.131*** (18.016)	0.134*** (18.454)	0.133*** (18.409)
*DEN*			-0.910*** (-1.907)	-0.945*** (-1.933)	-0.967*** (-2.036)	-0.980*** (-2.032)
*STR*				0.372*** (7.515)	0.353*** (7.190)	0.358*** (7.375)
*TTR*				-0.034*** (-4.213)	-0.028*** (-3.449)	-0.024*** (-3.025)
*FDI*					0.080*** (7.539)	0.078*** (7.393)
*ENV*					-0.264*** (-7.909)	-0.223*** (-6.661)
*SCI*						-0.043* (-1.823)
*EDU*						-0.102*** (-5.234)
*Constant*	0.187*** (251.135)	0.016 (0.856)	-0.078** (-2.193)	-0.181*** (-4.520)	-0.148*** (-3.636)	-0.128*** (-2.965)
Fixed	Yes	Yes	Yes	Yes	Yes	Yes
N	4 895	4 895	4 895	4 895	4 895	4 895
R^2^	0.888	0.896	0.905	0.909	0.911	0.912

Note: Standard errors in all regressions are robust standard errors for urban time clustering; t values for parameter estimates are in parentheses;

***, **, * represent statistical significance at the 1%, 5%, and 10% levels, respectively, controlling for year and individual fixed effects, as shown below.

### 5.2 Robustness

To ensure that the conclusions obtained are robust, we conduct four robustness tests. The results are shown in Table 3.

**Table 3:** Robustness tests.

Variables	Replacement of pollutants			Excluding municipalities	Excluding exceptional years
	(1)	(2)	(3)	(4)	(5)
	Net CO2	PM2.5	Dust	CO2	CO2
*EIO*	-0.269** (-2.449)	-0.031*** (-2.589)	-0.031*** (-2.680)	-0.327*** (-3.384)	-0.314*** (-3.215)
Constant	Yes	Yes	Yes	Yes	Yes
N	4 895	4 895	4 895	4 827	4 321
R^2^	0.950	0.670	0.680	0.910	0.906

Note: All regression equations control for time and individual fixed effects. Expressions are omitted to streamline the table, and constant term regression coefficients are omitted to show the same below.

#### 5.2.1 Tests on other pollutants

Considering the dynamic balance of CO_2_ emissions and CO_2_ sequestration, this study first measures the CO_2_ sequestration value of terrestrial vegetation and then obtains the net CO_2_ emissions by subtracting the CO_2_ sequestration value from the CO_2_ emissions. This measure is used as a robustness test. The results are shown in column (1) of Table 3. The average concentration of fine particulate matter (PM_2.5_) plus industrial smoke and dust emissions are also used as proxies for pollution at the level of prefecture-level cities. The regression results are shown in columns (2) and (3) of Table 3. The analysis results show that the regression coefficients of the import openness to environmental intermediate goods are significantly negative regardless of whether net CO_2_ emissions or the average PM_2.5_ concentration and industrial smoke and dust emissions are used as urban environmental measures, which is consistent with the baseline regression results. This finding indicates that environmental intermediate goods imports reduce not only CO_2_ emissions but also other harmful gas emissions and improve urban environmental quality in all aspects.

#### 5.2.3 Excluding samples from municipalities

Considering that municipalities directly under the central government have a higher level of economic development, administrative status and openness to the outside world and enjoy more policy advantages, some of their measurement indicators cannot be generalized to ordinary prefecture-level cities. Therefore, to avoid interference from city-level factors, this study removes the sample of four municipalities directly under the central government. The results are shown in column (4) of Table 3. The analysis shows that after removing the effect of the municipalities, the obtained results are consistent, and the regression coefficient of the import openness to environmental intermediate goods is still significantly negative.

#### 5.2.4 Excluding the impact of the financial crisis

The outbreak of the financial crisis that swept the world in 2008 had a serious impact on international trade and economic activity. Given the downward pressure on the economy and the increased focus on economic growth, governments increased investment and moderately reduced environmental regulations. Considering the duration of this crisis, the study excludes the 2008 and 2009 samples to ensure the representativeness of the sample selection. The results are presented in column (5) of Table 3. The results show that the regression coefficient of the import openness to environmental intermediate goods remains significantly negative, which further confirms the robustness of the study results.

### 5.3 Endogeneity

The baseline regression in this paper may have endogeneity problems due to reverse causality and omitted variables that affect the empirical results. With regard to examining the causes, this study controls for the interference of individual and time effects, the level of economic development of prefecture-level cities, and environmental protection policies in the baseline regression. However, there may still be some variables that have not yet been captured, such as the geographical location, climate, development stage, and historical and cultural background of prefecture-level cities that affect regional CO_2_ emissions and openness to import environmental intermediate goods. Furthermore, the import of environmental intermediate goods and CO_2_ emissions may causally interact and influence each other. In cities with lower CO_2_ emissions, the government and the community pay more attention to the positive interaction between environmental protection and economic development, adopt regulatory measures such as environmental restrictions and penalties for illegal emissions, and introduce stricter regulations for industrial pollution control, which induce more enterprises to import environmental intermediate goods with green technologies and ultimately reduce CO_2_ emissions. To eliminate endogeneity effects, this study uses a dynamic panel model and instrumental variables (IVs) to further test the results. These results are shown in Table 4.

**Table 4:** Endogeneity treatment.

Variables	Difference GMM		System GMM		IV-2SLS
	(1)	(2)	(3)	(4)	(5)
	CO2	CO2	CO2	CO2	CO2
*EIO*	-0.961*** (-4.266)	-0.558*** (-2.537)	-0.652*** (-3.082)	-0.169 (-1.043)	-1.085*** (-3.639)
*L*.*CO2*	0.185*** (4.290)	0.218*** (4.326)	0.588*** (12.126)	0.628*** (14.737)	
*L*.*EIO*		-0.372*** (-2.715)		-0.566*** (-4.862)	
Control	Yes	Yes	Yes	Yes	Yes
R^2^					0. 676
AR(1) p value	0.000	0.000	0.000	0.000	
AR(2) p value	0.417	0.267	0.670	0.892	
Hansen value	242.302	259.248	238.700	265.883	
Hansen p value	0.232	0.213	0.300	0.642	
Kleibergen‒Paap rk LM and p value					104.330 (0.000)
Cragg-Donald Wald F and the10% threshold					586.335 [16.38]
N	4 299	4 299	4 595	4 595	4 594

#### 5.3.1 Dynamic panel model

Considering the high autocorrelation of CO_2_ emissions, i.e., current CO_2_ emissions may be affected by previous CO_2_ emissions and may exhibit inertia, we introduce the first-order lagged terms of the explanatory variables in the econometric model (19) to establish a dynamic panel data econometric model. This model also accounts for the highly autocorrelated nature of imports. Dynamic panel data econometric models mainly have two methods, difference generalized method of moments (GMM) and system GMM, and both methods are used for regression in this paper.

In Table 4, from the AR (1) test, AR (2) test and Hansen test p value, it is clear that the dynamic panel model is reasonably set up. On this basis, the first-order lag regression coefficient of CO_2_ emissions is significantly positive, which verifies that CO_2_ emissions have continuity. The regression coefficients of both the import openness to environmental intermediate goods and its first-order lagged term are significantly negative, which after excluding the inertia of CO_2_ emissions, indicates that the influence of environmental intermediate goods imports has long-term stability.

#### 5.3.2 Instrumental variable approach

Column (5) of Table 4 is the result of using a one-period lag of the import openness to environmental intermediate goods at the level of prefecture-level cities as the IV in the IV two-stage least squares (2SLS) estimation method. The regression coefficient of the import openness to environmental intermediate goods remains consistent with the baseline regression result and is still significantly negative. This result indicates that the conclusion that environmental intermediate goods imports can reduce CO_2_ emissions still holds after eliminating the endogeneity problem caused by possible reverse causality.

### 5.4 Heterogeneity

#### 5.4.1 Heterogeneity of geographical location

To study the heterogeneous effects of the import openness to environmental intermediate goods on CO_2_ emissions under different geographical locations, this study divides the cities into the eastern, central, and western regions of China and conducts separate regressions. The regression results are shown in columns (1) to (3) of Table 5. The results in Table 5 show that the significance and estimated values of the regression coefficients of the import openness to environmental intermediate goods keep decreasing from Eastern China to Central China to Western China, indicating that the effect keeps decreasing from east to west. This finding is mainly due to the eastern region’s geographical advantage of proximity to the coastline and the policy advantage of reform and opening up compared to the central and western regions, resulting in greater import competition. In addition, the degree of industrialization maturity and market competition in the eastern region is higher than that in the central and western regions. The larger the scale of imports is, the more intense the import competition, forcing enterprises to accelerate technological innovation, improve resource utilization efficiency to maintain competitive advantage, and ultimately improve enterprise productivity and reduce pollutant gas emissions. The central and western regions are far from ports and have low import convenience, and for the economy to develop, they must take on many high-energy-consuming industries that have transferred from the eastern region.

**Table 5:** Heterogeneity of geographical location and the contamination level.

Variables	Geographical location			Contamination level	
	(1)	(2)	(3)	(4)	(5)
	Eastern	Central	Western	Severe	Less
*EIO*	-0.672*** (-3.407)	-0.251 (-1.641)	-0.125 (-0.917)	-1.061*** (-5.272)	-0.126* (-1.916)
Control	Yes	Yes	Yes	Yes	Yes
N	1 842	1 693	1 360	2 419	2 476
R^2^	0.922	0.882	0.930	0.913	0.846

#### 5.4.2 Heterogeneity of the contamination level

To investigate the heterogeneous effects of the import openness to environmental intermediate goods on CO_2_ emissions under different degrees of environmental pollution, this study uses the annual average value of CO_2_ emissions as a threshold and divides the prefecture-level cities into severely polluted cities and less polluted cities for the regressions. The corresponding results are presented in columns (4) and (5) of Table 5. The absolute value of the regression coefficient of the import openness to environmental intermediate goods in severely polluted cities is significantly higher than that in less polluted cities, which indicates that the effect is more obvious in severely polluted areas. The reason for this finding is that most heavily polluting cities have a heavy industrial base and the ability to absorb and transform green technologies into imported products. They are in the industrial transformation stage, and since the government attaches importance to stricter environmental regulations, the effect of importing and reducing emissions from environmental intermediate goods is stronger than that in less polluted prefecture-level cities.

#### 5.4.3 Heterogeneity of sustainable development ability

Considering the differences in development patterns and resource endowments, there are significant differences in sustainable development ability between prefecture-level cities. On this basis, this paper draws on existing studies to classify cities into two groups, high and low, based on their sustainability capacity, and it conducts separate regressions [25]. The regression results are shown in columns (1) to (2) of Table 6. The results in Table 6 show that the higher the sustainability of a city is, the more pronounced the abatement effect of the import of environmental intermediate goods. The possible reason for this result is that the more sustainable a city is, the more open it is to the import of high-quality intermediate goods, and the more trade acts as a technology transfer channel. The stronger the sustainability of a city is, the greater the environmental protection and pollution reduction efforts, and the higher the enthusiasm for the import of environmental intermediate goods. The complementary coordination between different products increases the green production capacity of enterprises, prompting them to integrate into the international division of labor system at a higher level and strengthen their own green sustainable development capacity.

**Table 6:** Heterogeneity analysis of sustainability and government efficiency.

Variables	Sustainable development ability		Government efficiency	
	(1)	(2)	(3)	(4)
	High	Low	High	Low
*EIO*	-0.789*** (-5.064)	0.031 (0.271)	-0.708*** (-4.388)	-0.056 (-0.476)
Control	Yes	Yes	Yes	Yes
N	3 276	2 519	2 458	2 437
R^2^	0.912	0.909	0.917	0.909

#### 5.4.4 Heterogeneity of government efficiency

A good business environment and market order are important prerequisites for the survival and development of enterprises. Hence, government efficiency is the key to influencing the emission reduction effect of environmental intermediate imports. On this basis, this paper divides prefecture-level cities into two groups, high and low, based on city government efficiency, groups them for regression, and conducts separate regressions [25]. The regression results are shown in columns (3) to (4) of Table 6. The results in Table 6 show that the higher the government efficiency of a city is, the more pronounced the abatement effect of the import of environmental intermediate goods. The possible reason for this result is that the higher the efficiency of the government, the timelier and more effective it is in rectifying the problems revealed by the auditing authorities, such as imperfect low-carbon policy measures, inadequate implementation and ineffectiveness. Improved government efficiency can avoid problems such as low-carbon fiscal funds not being allocated as needed, irregularities in declaration management and use, lack of integration and use, and ineffective use. An improvement in government efficiency helps to promote the safe and efficient use of low-carbon financial funds and the accelerated promotion and efficient operation of low-carbon engineering projects. Therefore, the higher the efficiency of the government is, the more obvious the emission reduction effect of environmental intermediate imports.

### 5.5 Mechanism testing

#### 5.5.1 Green technological innovation

To verify hypothesis 2, we measure the green science and technology innovation (*GTEC*) of prefecture-level cities by the ratio of the number of green utility patent applications to the total number of utility patent applications in the year [26] based on data from the Chinese patent database. On this basis, this paper analyzes whether the import of environmental intermediate goods can promote green technological innovation. The estimation results are presented in column (1) of Section 7.

An analysis of the regression results shows that the coefficient of EIO is significantly negative, which indicates that imports of environmental intermediate goods will cause spillovers of green technology and promote local imitation and learning of advanced technology. This finding validates hypothesis 2. Trade openness attracts more environmental products into the local market and intensifies market competition, thus forcing enterprises to strengthen R&D, improving clean technology R&D to enhance market competitiveness, enhancing the final treatment capacity for pollution emissions, and ultimately improving environmental quality. In addition, given the continuous development of green development, each prefecture-level city pays more attention to its own environmental responsibilities and obligations in the development process and pays attention to financial investment and policy support for science and technology innovation, which enables green technology R&D activities and ultimately promotes local green technological innovation and CO_2_ emission reductions.

#### 5.5.2 Upgrading the industrial structure

To test hypothesis 3, this study uses the coefficient of industrial structure upgrading (*STRUP*) to measure industrial structure upgrading [27]. The interaction term between the coefficient of industrial structure upgrading and the import openness to environmental intermediate goods is introduced into the regression model to investigate whether imports of environmental intermediate goods can reduce CO_2_ emissions by promoting the upgrading of the industrial structure. The estimation results are shown in column (2) of Table 7.

**Table 7:** Mechanism tests.

Variables	Green Technological Innovation	Industrial Structure Upgrading
	(1)	(2)
	*GTEC*	*STRUP*
*EIO*	0.210** (2.273)	0.292*** (2.785)
Control	Yes	Yes
N	4 895	4 895
R^2^	0.283	0.812

The coefficient of the interaction term is significantly negative, indicating that imports of environmental intermediate goods can address environmental pollution by promoting the upgrading of the industrial structure. This finding validates hypothesis 3. The reason for this result is that with the continuous increase in imports of environmental intermediate goods, the concept of green development has been promoted, which increases the consumption demand of residents for low-carbon final goods and drives the development and growth of related industries, thus accelerating industrial structure upgrading and reducing CO_2_ emissions. Moreover, the increase in imports intensifies market competition, accelerates the process of eliminating industry winners and losers, and triggers the process of optimizing the reallocation of resources at the industry and enterprise levels. This process not only continuously compresses the survival space of low-end enterprises and drives out polluting enterprises but also promotes the continuous transfer of resources from low-end industries to high-end industries, ultimately improving the coupling degree of green input and output, developing and growing green and low-carbon industries, and reducing pollutant emissions.

## 6. Conclusions and policy recommendations

### 6.1 Research conclusions

The importation of environmental intermediate goods creates not only an important hub to promote the development of international trade and deep participation in the global value chain but also an important means by which to share technological achievements and achieve global green and low-CO_2_ development. However, theoretical models to support research on how imports of environmental intermediate goods affect regional CO_2_ emissions are lacking. Thus, the spatial spillover effect of imports of environmental intermediate goods on CO_2_ emissions has not been effectively verified. Hence, this study constructs a biased production decision model of enterprises based on imports of environmental intermediate goods to clarify how imports of these goods affect enterprises’ production decisions and, thus, their final CO_2_ emissions at the theoretical level. Meanwhile, this paper empirically verifies the theoretical conclusions based on data on the import trade in environmental intermediate goods and data on CO_2_ emission pollution in Chinese prefecture-level cities from 2000 to 2016. The main research conclusions are as follows:

First, from the analysis of the theoretical model and empirical analysis, this paper found that the import of high-quality environmental intermediate goods can effectively play a role in carbon emission reduction. This conclusion is robust and effective after expanding the sample range of imported intermediate goods, replacing the measurement indicators, eliminating possible sampling bias and years, and eliminating potential endogenous impacts. The expansion of the import scale of environmental intermediate goods can change the direction of production decisions and technological progress of enterprises and expand the R&D of green low-carbon technologies, thus effectively reducing CO_2_ emissions.

Second, imports of environmental intermediate goods can effectively reduce regional CO_2_ emissions, and green technology R&D and industrial structure upgrading play an important role in the transmission of this effect. The innovation effect of green technology not only reduces the emission of pollutants in the production process but also enhances the final treatment capacity for pollutants. The effect of industrial structure upgrading accelerates the process of eliminating industry winners and losers, leading to the withdrawal of polluting enterprises, compressing the survival space of backward industries, and ultimately promoting the optimal allocation of resources and the development and growth of green industries.

Finally, the emission reduction effect of importing environmental intermediate goods has obvious heterogeneity and is influenced by factors such as geographical location, environmental pollution, sustainable development ability and government efficiency. The carbon reduction effect of imports of environmental intermediates is more pronounced in cities in the eastern region with severe pollution, higher sustainable development ability and more efficient governments.

### 6.2 Policy recommendations

First, the government should appropriately reduce tariffs on specific environmental intermediate goods and strive to improve conditions for import trade liberalization and facilitation. It should accelerate the optimization of import customs clearance links, realize speed and fee reduction, and better facilitate imports of foreign environmental intermediate goods.

Second, the government should effectively undertake policy and improve administrative efficiency, actively promote the revision of product energy consumption limits and mandatory national standards for product and equipment energy efficiency, reduce and eliminate barriers to the trade in environmental intermediate goods, and promote the cross-border flow of environmental intermediate goods to jointly achieve low-carbon development.

Third, the government should build an industry-university-research cooperation platform, enhance the capacity of local enterprises for independent innovation and scientific R&D, promote the transnational exchange of low-carbon technologies, promote the optimal allocation of scientific research resources and resource sharing, make the R&D of new technologies compatible with the new national green development concept, and continuously promote the transformation of scientific and technological achievements into productivity.

### 6.3 Limitations and further research

The empirical analysis in this paper is based on the influence of environmental intermediate goods imports on CO_2_ emissions in prefecture-level cities in China, but the sample years used in this paper are from 2000 to 2016 because the latest data from the China customs database are current only up to 2016, thus limiting the data for environmental intermediate goods imports in prefecture-level cities. Although these panel data are long enough to illustrate and test the questions we want to study, they may be somewhat less time sensitive, and further research can be conducted in the future as updated yearly data become available. Meanwhile, due to the limitation of data availability, there are currently no carbon emission data at the firm level. Therefore, the theoretical analysis is mainly based on the firm level, while the empirical analysis is mainly based on the city level. Future research can consider further investigation from the perspective of upstream and downstream production chains and the production networks of firms.
